# Discrimination of Cancer Stem Cell Markers ALDH1A1, BCL11B, BMI-1, and CD44 in Different Tissues of HNSCC Patients

**DOI:** 10.3390/curroncol28040241

**Published:** 2021-07-19

**Authors:** Kariem Sharaf, Axel Lechner, Stefan P. Haider, Robert Wiebringhaus, Christoph Walz, Gisela Kranz, Martin Canis, Frank Haubner, Olivier Gires, Philipp Baumeister

**Affiliations:** 1 Department of Otolaryngology, University Hospital, LMU Munich, 81377 Munich, Germany; Axel.Lechner@med.uni-muenchen.de (A.L.); Stefan.Haider@med.uni-muenchen.de (S.P.H.); Robert.Wiebringhaus@med.uni-muenchen.de (R.W.); Gisela.Kranz@med.uni-muenchen.de (G.K.); Martin.Canis@med.uni-muenchen.de (M.C.); Frank.Haubner@med.uni-muenchen.de (F.H.); Olivier.Gires@med.uni-muenchen.de (O.G.); Philipp.Baumeister@med.uni-muenchen.de (P.B.); 2 Institute of Pathology, Faculty of Medicine, LMU Munich, 81377 Munich, Germany; Christoph.Walz@med.uni-muenchen.de; 3 Clinical Cooperation Group “Personalized Radiotherapy in Head and Neck Cancer”, Helmholtz Zentrum München, 85764 Neuherberg, Germany

**Keywords:** CSCs, histopathology, HNSCC, tumor, lymph node, mucosa

## Abstract

Cancer stem cells (CSCs) are accountable for the progress of head and neck squamous cell carcinoma (HNSCC). This exploratory study evaluated the expression of molecular CSC markers in different tissues of HNSCC patients. Tissue specimens of primary tumor, lymph node metastases and macroscopically healthy mucosa of 12 consecutive HNSCC patients, that were treated with surgery and adjuvant radio(chemo)therapy upon indication, were collected. Samples were assessed for the expression of p16 as a surrogate for HPV-related disease and different molecular stem cell markers (ALDH1A1, BCL11B, BMI-1, and CD44). In the cohort, seven patients had HPV-related HNSCC; six thereof were oropharyngeal squamous cell carcinoma. While expression of BMI-1 and BCL11B was significantly lower in healthy mucosa than both tumor and lymph node metastasis, there were no differences between tumor and lymph node metastasis. In the HPV-positive sub-cohort, these differences remained significant for BMI-1. However, no significant differences in these three tissues were found for ALDH1A1 and CD44. In conclusion, this exploratory study shows that CSC markers BMI-1 and BCL11B discriminate between healthy and cancerous tissue, whereas ALDH1A1 and CD44 were expressed to a comparable extent in healthy mucosa and cancerous tissues.

## 1. Introduction

Head and neck squamous cell carcinoma (HNSCC) is among the most prevalent cancers worldwide [1]. Risk factors include the consumption of tobacco, alcohol, and betel nut and exposure to human papillomavirus (HPV) [2]. Of the mentioned risk factors, high-risk HPV, e.g., HPV 16, 18, and 33, cause an increasing fraction of HNSCC incidence worldwide, and mortality is relatively constant in recent years [3,4,5]. In addition to TNM classification, resection margins status, extracapsular extension, and lymphovascular invasion, HPV status can predict survival probabilities in oropharyngeal squamous cell carcinoma, which eventually led to a new TNM classification system for p16-positive oropharyngeal carcinomas [6,7,8,9].

To overcome stagnation in the prognosis of HNSCC, research on the identification of prognostic markers and therapeutic targets is crucial and prominently called for [10]. Cancer stem cells (CSCs; also known as tumor-initiating cells) are considered important targets in this context. Though CSCs are believed to foster HNSCC progression and recurrence, representing a dormant cancer cell type, more resistant to non-surgical therapy such as radiotherapy and chemotherapy [11], their identification remains challenging [12].

In HNSCC, ALDH1 (Aldehyde dehydrogenase 1), BMI-1 (B-lymphoma Moloney murine leukemia virus insertion region-1), and CD44 belong to the most studied CSC markers and, additionally, BCL11B (B-cell lymphoma/leukemia 11B) was found to be a CSC marker in strong co-expression to BMI-1 [13,14,15]. ALDH1 is important for the maintenance of stemness and differentiation of stem cells [16]. BMI-1 plays a role in the self-renewal ability of stem cells, and high expression in cancer was related to epithelial-mesenchymal transition (EMT) and poor prognosis [13,17]. CD44 is a surface glycoprotein and common CSC marker in several human tumor entities [18] whose high expression was associated with a negative prognosis in oral cancer [19]. BCL11B is linked to both embryogenesis and tumor suppression [13,14]. In a previous study of this group, the correlation of CSC marker expression and prognosis of survival was studied. In a cohort of advanced-stage HNSCC patients treated with primary radio(chemo)therapy, CSC markers had a differential influence on survival, while HPV status had no significant influence. Of the CSC markers, BMI-1 and CD44 indicated a significantly worse prognosis on survival, while ALDH1 and BCL11B appear to have protective properties for HNSCC survival [20].

Moreover, targeting CSCs specifically in therapeutical approaches is highly challenging because potential molecular target sites bear risks of cross-reactions and toxicity. For example, the development of an anti-CD44 immunoconjugate for therapeutic use in patients with advanced HNSCC was terminated after the experimental treatment resulted in skin toxicity [21,22].

In an exploratory approach, we searched for differences in the expression of these CSC markers in primary tumor tissue, lymph node metastases, and healthy mucosa in patients with HNSCC.

## 2. Materials and Methods

### 2.1. Patients

In this single-center prospective exploratory study, we included 12 consecutive patients who were (a) diagnosed with head and neck squamous cell carcinoma, (b) treated with surgical tumor resection and neck dissection, (c) whose macroscopically healthy mucosa was biopsied after written informed consent, and (d) whose tumor and lymph node specimens were positive for squamous cell carcinoma.

Clinical data, including risk factors such as smoking and drinking habits and staging, were obtained from the patients’ medical records. Histologic samples were collected and analyzed as described below. Detailed patient characteristics are provided in Table 1. All data is displayed for the entire sample set regardless of the HPV status and separately for the HPV-distinct sub-cohorts.

All clinical samples were obtained with written informed consent during routine surgery or biopsy based on the approval by the ethics committee of the local medical faculty (Ethikkommission der Medizinischen Fakultät der Ludwig-Maximilians-Universität, IRB approval number 18-446) and in compliance with the WMA Declaration of Helsinki.

### 2.2. Histological Samples and Immuno-Staining

We obtained 8.0 mm diameter punch biopsies from the primary tumor after resection and macroscopically healthy mucosa. Healthy mucosa was taken beyond tumor-negative margins, proven by intraoperative frozen sections. One lymph node of the neck dissection specimen that was macroscopically suspicious of metastasis was taken. All specimens were embedded in tissue-tek (Sakura, Fintek, The Netherlands) and snap-frozen in liquid nitrogen before processing to 4 µm thick consecutive cryosections. Antigen expression was assessed in 3-4 tissue slides. P16 status as a surrogate marker of HPV infection will be referred to as HPV status in the following (p16-specific antibodies: cs56330, Santa Cruz Biotechnology) [23,24]. Immunohistochemistry (IHC) was performed using ALDH1A1-, BCL11B-, BMI-1-, and CD44-specific antibodies (ALDH1A1 1:100: ab9883, abcam, Cambridge, MA, USA; BCL11B 1:500: ab18465, abcam, Cambridge, MA, USA; BMI-1 1:100: ab14389, abcam, Cambridge, MA, USA; CD44 1:500: VP-C353, Vector Laboratories, Burlingame, CA, USA). The avidin-biotin-peroxidase complex method (Vectastain, Vector laboratories, Burlingame, CA, USA) was used according to the manufacturer’s protocol. Sections were incubated with the respective primary antibody for 1 h at room temperature (RT) followed by incubation with biotinylated anti-mouse immunoglobulins and then avidin-biotin-peroxidase complex (30 min at RT for each step). After each step, sections were washed with PBS. Specific peroxidase activity was visualized with 0.05% 3-amino-9-ethylcarbazol (Sigma, Deisenhofen, Germany) and 0.02% H_2_O_2_/0.1M Na-acetat buffer pH 5.5 as a substrate. Counterstaining was performed with hematoxylin. As a negative control, staining was performed with mouse pre-immune serum instead of specific antibodies.

As a histopathological reference regarding the immunoexpression of the markers in formalin-fixed, paraffin-embedded samples, we compared samples of this manuscript to the HNSCC tumor tissue micro assays of the Göttingen cohorts of our former manuscript [20]. For quality control reasons and to identify tumor infiltrates in the HNSCC and LN tissues, we performed hematoxylin/eosin stainings of the samples without the IHC markers as well as control stainings with mouse pre-immune serum and the IHC secondary antibodies alone instead of specific antibodies and secondary antibodies to exclude unspecific staining. No specific staining in the tumor tissue or mucosa was found in these controls.

In order to quantify and compare expression levels, immunohistochemistry scores (IHC scores) were applied to all staining. IHC scores represent the product of the percentages of positive cells, and the staining intensity scored from negative (0), low (1), intermediate (2), to strong (3). Thus, scores range from 0 to 300 and are referred to as H-scores as described before [25]. Four experienced examiners performed the analysis independently, and scores were consented.

### 2.3. Statistical Analysis

Since results from the primary tumor, healthy mucosa, and lymph node metastasis of each individual patient are interconnected, IHC score data were analyzed by either one way repeated measures (RM) analysis of variance (ANOVA) with the Geisser–Greenhouse correction and Tukey’s multiple comparisons test for the entire sample set regardless of the HPV status or two way RM ANOVA with the Geisser-Greenhouse correction and Tukey’s multiple comparisons test separately for the HPV-distinct sub-cohorts. A *p*-value < 0.05 was considered statistically significant. All statistical procedures were conducted using GraphPad Prism 9.1.0 (GraphPad Software, San Diego, CA, USA).

## 3. Results

### 3.1. Study Cohort

All 12 patients in the cohort received surgical treatment with or without adjuvant radiotherapy. Seven patients had p16-positive HNSCC (hereafter referred to as HPV-positive or HPV+), and five patients had p16-negative HNSCC (hereafter referred to as HPV-negative or HPV−). Out of seven patients with HPV-positive HNSCC, six had oropharyngeal squamous cell carcinoma. Detailed patient characteristics, risk factors, and disease staging are shown in Table 1, as stated in the methods section.

### 3.2. Quantitative Analysis of Immunohistochemical Scorings

First, to explore differences in the expression of CSC markers, the expression of a single CSC marker was compared between normal mucosa, primary tumor, and nodal metastases. After that, CSC marker expression was further compared between the three different tissues for the HPV-distinct sub-cohorts. The marker expressions within the same kind of tissue between HPV-positive and HPV-negative cases were compared.

**Figure 1:**
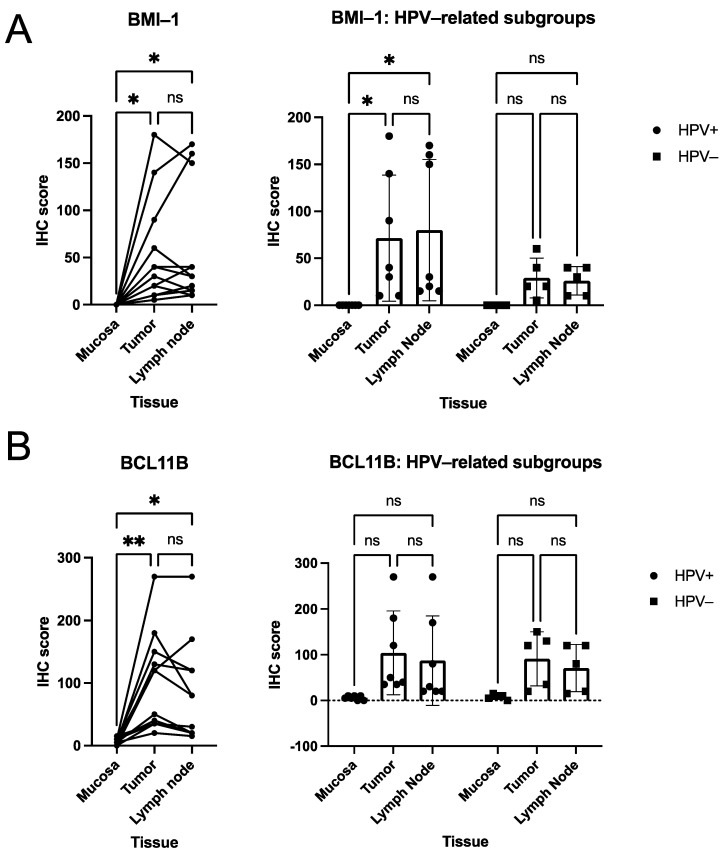
Quantitative analyses of BMI-1 (**A**) and BCL11B (**B**) IHC scores in HNSCC. Connected dots deflect IHC scores of one individual patient in the exploratory analysis of HNSCC patients. Plots show means with SD of data stratified according to HPV-status (HPV-positive versus HPV-negative). *p*-values (one way RM ANOVA for cohort analyses, two-way RM ANOVA for subcohort analyses) are indicated (* *p* < 0.05, ** *p* < 0.01, n.s. *p* > 0.05).

In the cohort, BMI-1 expression (Figure 1A) was significantly higher in both tumor (Tu) and lymph node metastasis (LN) than in healthy mucosa (Muc), whereas no differences were observed between tumor and lymph node (Tu vs. Muc *p* = 0.017, LN vs. Muc *p* = 0.022, Tu vs. LN *p* = 0.887; one way RM ANOVA; median H-scores: Muc 0, Tu 35, LN 30). In the HPV-positive sub-cohort, similar results were observed (HPV+ Tu vs. respective Muc *p* = 0.016, HPV+ LN vs. respective Muc *p* = 0.016, HPV+ Tu vs. HPV+ LN *p* = 0.182; two way RM ANOVA). All comparisons in the HPV-negative sub-cohort and comparisons between the HPV-distinct tumor samples, lymph node metastasis samples, and respective mucosa samples did not attain statistical significance (HPV− Tu vs. respective Muc *p* = 0.061, HPV- LN vs. respective Muc *p* = 0.059, all other *p* > 0.05; two way RM ANOVA).

Concordantly, BCL11B expression (Figure 1B) was significantly higher in both tumor and lymph node metastasis than in healthy mucosa. In contrast, no differences between tumor and lymph node were observed (Tu vs. Muc *p* = 0.004, LN vs. Muc *p* = 0.021, Tu vs. LN *p* = 0.197; one-way RM ANOVA; median H-scores: Muc 7.5, Tu 85, LN 55). In the HPV-distinct cohorts, neither between tissues nor between tumor, lymph node metastasis, nor respective mucosa samples (HPV-positive subcohort vs. HPV-negative subcohort) significant differences were found (all *p* > 0.05; two way RM ANOVA).

**Figure 2:**
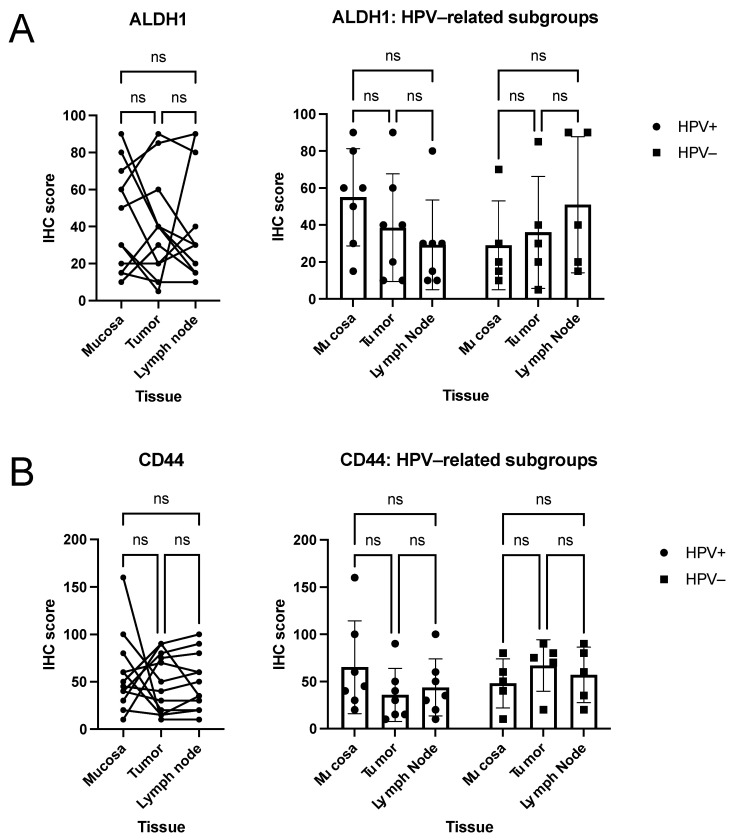
Quantitative analyses of ALDH1 (**A**) and CD44 (**B**) IHC scores in HNSCC. Connected dots deflect IHC scores of one individual patient in the exploratory analysis of HNSCC patients. Plots show means with SD of data stratified according to HPV-status (HPV-positive versus HPV-negative). *p*-values (one-way RM ANOVA for cohort analyses, two-way RM ANOVA for subcohort analyses) are indicated (n.s. *p* > 0.05): No significant differences were detected in the exploratory analyses of ALDH1 and CD44.

In contrast to BMI-1 and BCL11B, no differential expression was found for ALDH1 and CD44 (Figure 2A,B) in the cohort (all *p* > 0.05; one way RM ANOVA; median H-scores ALDH1: Muc 40, Tu 35, LN 30; median H-scores CD44: Muc 47.5, Tu 45, LN 42.5). Similarly, IHC scores did not differ significantly between tissues in the HPV-distinct sub-cohorts (all *p* > 0.05; two-way RM ANOVA).

### 3.3. Descriptive (Microscopical) Findings in the Staining for Molecular Markers of Interest

**Figure 3:**
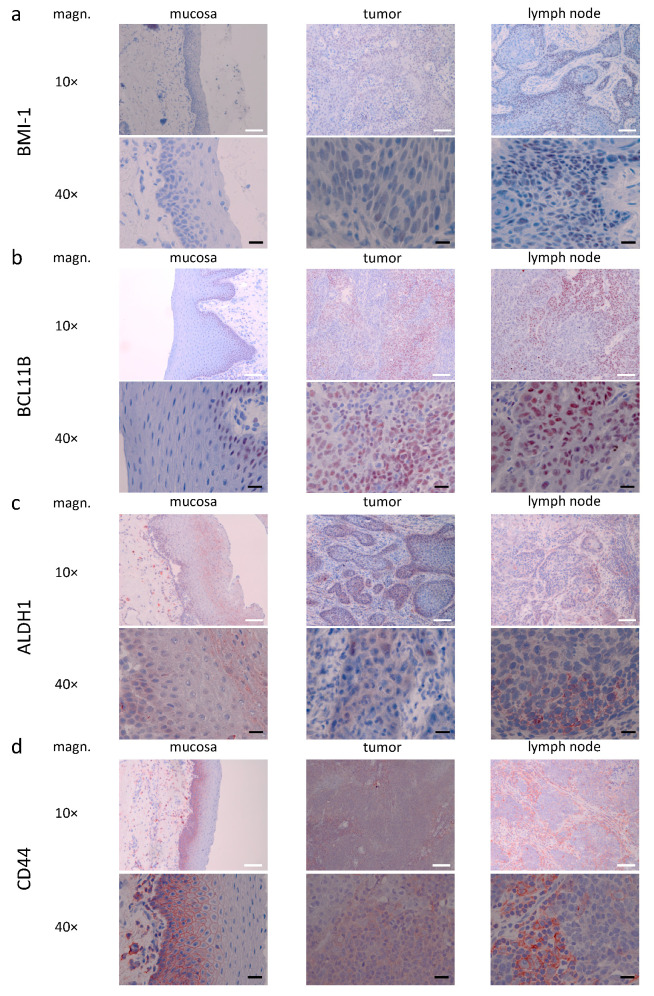
Expression patterns of CSC markers ALDH1, BCL11B, BMI-1, and CD44 in the primary tumor, lymph node metastasis, and healthy mucosa. Shown are representative examples of BMI-1 (**a**), BCL11B (**b**), ALDH1 (**c**), and CD44 (**d**) expression (10× and 40× magnification) in HNSCC. The white bar in 10× magnification equals 100 µm, the black bar in 40× magnification equals 20 µm. Samples from the cohort with median IHC scores regardless of HPV status were chosen. ALDH1, BCL11B, BMI-1, and CD44 staining is red-brown, nuclei and cytoplasm are counter-stained with hemalaun (blue).

For the following microscopical characterization, patient samples with median IHC scores from the conducted analyses were chosen (Figure 3). Examples of strong marker expression within this cohort are depicted in Figure S1.

A representative for BMI-1, IHC shows nuclear staining within HNSCC cells and neither staining in lymphocytes nor vessels nor connective tissue. Additionally, slight cytoplasmatic staining was seen. Within metastatic lymph node tissue, the same pattern of staining appeared. In mucosa tissue, no mucosa-specific staining was seen (Figure 3a).

Showing nuclear IHC stainings, BCL11B was regularly seen in HNSCC cells and immune cells. The same staining pattern was found in lymph node metastases. Except for light staining in the *stratum basale*, no staining was seen in mucosa tissue (Figure 3b).

In contrast, ALDH1 expression was diffuse and mostly found in the cytoplasm of HNSCC cells. In lymph node metastases, similar staining was seen in HNSCC cells and sporadic staining in immune cells. Interestingly, within mucosal tissue, cytoplasmatic staining was found in the *stratum basale* and *stratum granulosum*, but less pronounced in the *stratum spinosum*. In addition, staining occurred in vessels and sporadic cells within the mucosal *lamina propria* (Figure 3c).

In the primary tumor tissue, staining for CD44 occurred in the cytoplasm of HNSCC cells and immune cells. In lymph node tissue, similar staining was seen in HNSCC cells and intensive cytoplasmatic staining in immune cells. Mucosal staining was seen in the *stratum basale* and *stratum spinosum*. Diffuse staining occurred in vessels, stromal and immune cells within the connective tissue. Compared to HNSCC cells, staining in *stratum basale* and *stratum spinosum* was more distinct in the cell membrane (Figure 3d).

## 4. Discussion

This study aimed to investigate the differential expression of CSC markers in HNSCC primary tumors, lymph node metastases, and macroscopically healthy mucosa of 12 consecutive patients with HPV-positive or -negative cancers identified using p16 staining [23,24]. The CSC carcinogenesis theory postulates that a small sub-group of cancer cells may predominantly perpetuate tumor growth, while most other cells possess little to no tumorigenic capability [26]. CSCs are believed to play a crucial role in resistance to treatment and tumor relapse: While treatment may effectively reduce tumor volume and induce a clinical response by decimating populations of differentiated cancer cells, CSCs may emerge unscathed and initiate tumor progression and, ultimately, treatment failure [26,27]. Hence, CSCs represent a potential target for new therapies [27].

As survival rates in HNSCC patients have shown little improvement over the past decades, novel approaches in treatment development and precision diagnostics and prognostication are paramount for improving outcomes. *Omics*-research, based on quantitative analysis of large sets of biologic data, yielded promising results [28,29,30]. However, CSCs, which represent only a small population of HNSCC cells, may escape wholistic analysis approaches such as radiomics, genomics, or proteomics. Thus, further study and characterization of CSCs specifically appear crucial to advancing HNSCC treatment and outcome.

The discontinuation of a trial [22] of an anti-CD44v6-immunoconjugate due to skin toxicity, which targeted a CSC marker that is also expressed in the skin, highlights the importance of studying the differential expression of such markers in various tissues, which was the purpose of this study.

Our study demonstrated expression levels of polycomb complex protein BMI-1 differed in benign and malignant tissue. While tumor and metastatic lymph node IHC scores were never significantly different, we found significantly higher scores in malignant tissue than benign mucosa in the combined cohort (Figure 1A). Analysis of the HPV-related and HPV-negative subgroups yielded similar results, with significantly higher IHC scores in malignant than benign tissue in the HPV-positive group and numerically but not significantly higher in the HPV-negative group (Figure 1A). The failure to attain significance in HPV-negative subjects may be related to a lack of statistical power. The potential role of BMI-1 in HNSCC outcome prognostication remains somewhat ambiguous at this time, with some studies supporting an association of BMI-1 expression with the poorer outcome or surrogate endpoints thereof and treatment response. In contrast, others failed to produce evidence supporting this notion [31,32,33,34]. A previous study by our group linked BMI-1 expression to reduced survival in the primary radio(chemo)therapy cohort. Still, no association was found in a cohort that underwent surgery as primary treatment [20]. This study, however, demonstrated strong differential BMI-1 expression, with markedly higher IHC scores in malignant tissues. In healthy mucosa, no mucosa-specific staining was identified (Figure 3). Our findings highlight the potential role of BMI-1 as an IHC marker for HNSCC detection and, to a lesser extent, for CSC detection.

Given its robust association with BMI-1, BCL11B has been proposed as CSC marker for HNSCC [14]. Indeed, our findings with respect to BCL11B expression were similar to BMI-1. High IHC-scores were observed in malignant tissues and significantly lower scores in the benign mucosa of the combined cohort (Figure 1B). BCL11B expression did not differ significantly between primary tumor and lymph nodes (Figure 1B). All these tendencies were retained in HPV-positive and -negative sub-groups, where our analysis again did not produce significant *p*-values, most likely due to a lack of power (Figure 1B). Similar to BMI-1, our results indicate that BCL11B may be utile in IHC-based HNSCC detection.

ALDH1, a family member of human aldehyde dehydrogenases, has received considerable attention within the CSC field [27]. Previous studies demonstrated a correlation of ALDH1 expression with metastatic spread to lymph nodes, increased tumor grade, size, and stage, and generally poorer outcome in HNSCC [13,35,36,37,38]. In oral cancer, ALDH1 is associated with higher tumor grade, lymph node metastases, angiolymphatic invasion, and resistance to treatment [27,39,40,41]. However, these findings are disputable to a certain extent, given that a previous study by our group could not identify a significant association of ALDH1 with survival in surgically treated HNSCC and even linked higher ALDH1 expression to a favorable outcome in patients who underwent primary radiochemotherapy [20]. At least one study investigated the potential of anti-ALDH1 small molecule targeted therapy—treatment with Aldi-6 resulted in higher sensitivity to cisplatin therapy in cell lines and restricted tumor growth in vivo [42]. Our study, however, revealed similar levels of ALDH1 expression in healthy mucosa and malignant tissues, with both mean and median mucosa IHC scores in HPV-associated HNSCC even surpassing that of tumors and metastases (Figure 2A). These findings may point to the increased potential for cisplatin-related adverse effects in mucosal tissue when combined with anti-ALDH1 treatment. In general, it highlights the pressing need for studies investigating the differential expression of target molecules in different tissues.

CD44 is a single chain transmembrane glycoprotein that acts as a receptor for hyaluronic acid [27] that has been identified as a specific marker for CSC in HNSCC [43,44,45]. In a study investigating CD44s and CD44v6 in head and neck mucosa, leukoplakia and carcinoma, the authors concluded expression levels of these molecules were not a suitable marker for differentiating malignant and pre-malignant tissue from normal tissue [46]. Our results substantiate these findings. IHC scores never differed significantly among tumoral, healthy mucosal, and metastatic tissues, neither in an analysis of the combined cohort nor in HPV sub-groups (Figure 2B). However, our prior study linked CD44 to poorer outcomes in patients treated with primary radiochemotherapy, while no such association was found in a surgical cohort [20]. These and other group findings emphasize the potential role CD44 may play in prognostication in patients who undergo radio- or chemotherapy [20,47,48,49,50,51,52].

Across all CSC markers investigated in this study, we demonstrated that expression levels were similar in primary tumors and metastatic lymph nodes. This finding supports the notion that CSC characteristics in metastasized cancer resemble those of the primary.

Our study was conducted in a relatively small cohort, which poses a potential limitation. Additionally, only the head and neck mucosa acted as a benign comparator tissue in assessing CSC marker differential expression. Further studies in larger cohorts are needed to confirm our findings, assess CSC marker expression in other relevant tissues, and investigate the differential expression of CSC markers in the context of HNSCC prognostication. Notably, identification of CSCs relied on single-CSC-marker IHC staining, and future studies may combine multiple markers in improving the specificity of CSC detection.

## 5. Conclusions

This exploratory study revealed no differential expression of CSC markers in primary tumors and lymph node metastases. While BMI-1 and BCL11B expression were higher in cancerous tissues than in healthy mucosa, no such differences were found for ALDH1 and CD44. These findings broaden the knowledge about CSC markers with respect to HNSCC. Most notably, BMI-1 and BCL11B might be utile in identifying CSCs in HNSCC IHC stainings.

## Figures and Tables

**Table 1:** General patient characteristics, risk factors, and disease stage of the surgical cohort. All continuous data variables are displayed as mean ± SD. HPV status is based on p16-immunostaining.

Characteristics		Cohort (*n* = 12)	HPV-neg. Subcohort (*n* = 5)	HPV-pos. Subcohort (*n* = 7)
**Sex**	Male Female	10 2	4 1	6 1
**Age at surgery [years]**		62.9 ± 6.8	59.3 ± 5.0	65.4 ± 7.1
**Relapse at diagnosis**	Positive	0	0	0
**Substance abuse**	Negative	3	0	3
	Nicotine (>10py)	7	4	3
	Alcohol	0	0	0
	Combined (Nicotine+Alcohol)	2	1	1
**Localization**	Oropharynx	9	3	6
	Hypopharynx	2	1	1
	Larynx	1	1	0
**UICC 8th edition staging**	pT 1	2	0	2
	2	3	1	2
	3	4	2	2
	4	3	2	1
	pN positive	12	5	7
	pN 1	7	1	6
	2	1	0	1
	2a	1	1	0
	3b	3	3	0
	ENE positive	8	3	5
	cM1	0	0	0
**Stage (UICC, 8th edition)**	I	4	0	4
	II	2	0	2
	III	1	1	0
	IVa	2	2	0
	IVb	3	2	1

## Data Availability

The data that support the findings of this study are available on request from the corresponding author.

## Supplementary Materials

The following are available online at https://www.mdpi.com/article/10.3390/curroncol28040241/s1, Figure S1: Examples of high expression patterns of CSC markers ALDH1, BCL11B, BMI-1, and CD44 in primary tumor, lymph node metastasis and healthy mucosa within the cohort.
