# Supplementary material for: Discrimination of Cancer Stem Cell Markers ALDH1A1, BCL11B, BMI-1, and CD44 in Different Tissues of HNSCC Patients
Source: Curr Oncol. 2021 Jul 19;28(4):2763–74. doi: 10.3390/curroncol28040241 (PMC8293237; doi:10.3390/curroncol28040241)
Supplement: Supplementary file 1 [file curroncol-28-00241-s001.zip › curroncol-1261875-supplementary.pdf]

Current Oncology

MDPI

Supplemental Material

Discrimination of Cancer Stem Cell Markers ALDH1A1, BCL11B, BMI-1, and CD44 in Different Tissues of HNSCC Patients

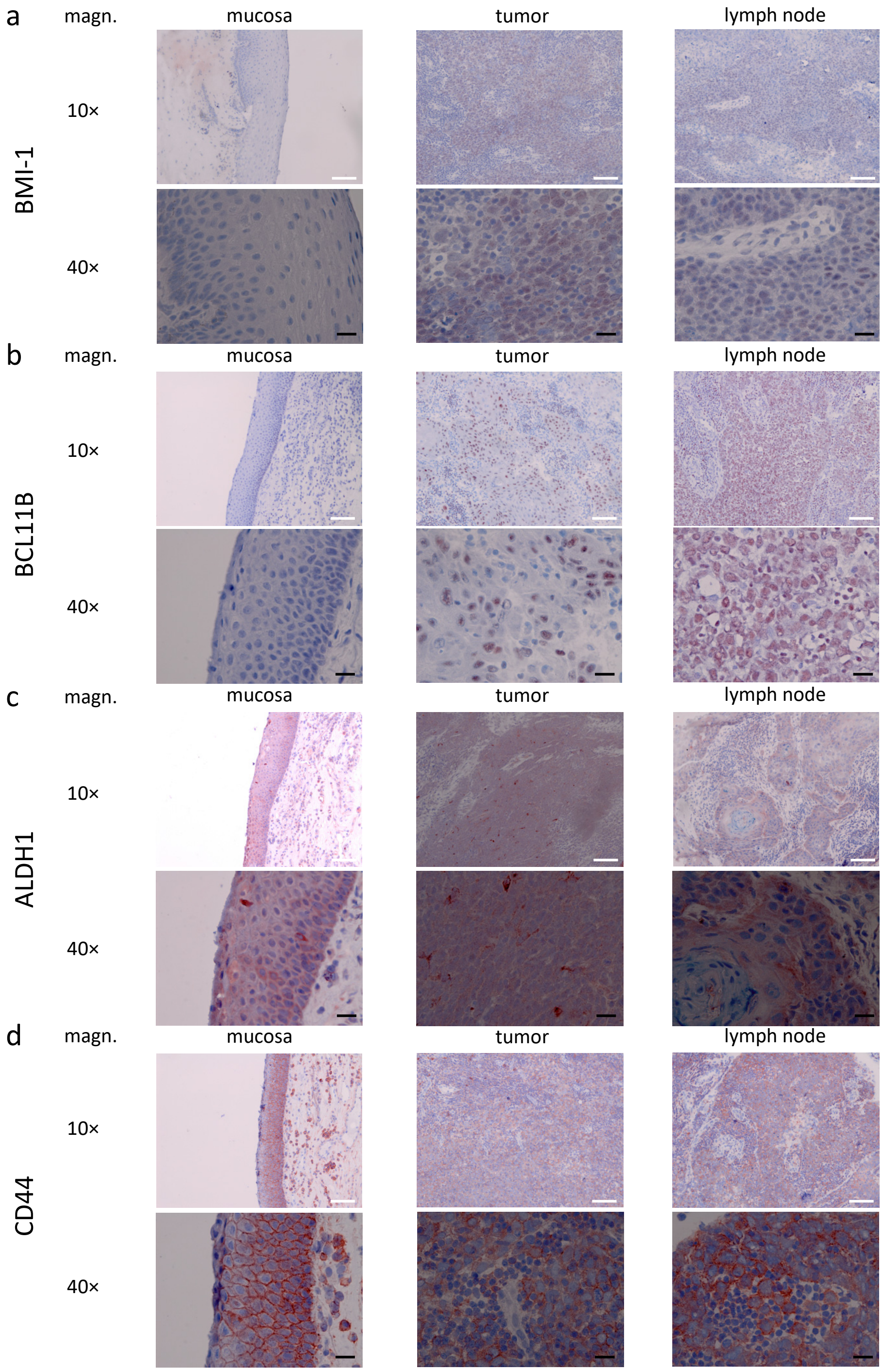

Figure S1. Examples of high expression patterns of CSC markers ALDH1, BCL11B, BMI-1, and CD44 in primary tumor, lymph node metastasis and healthy mucosa within the cohort. Shown are representative examples of BMI-1 (**a**), BCL11B (**b**), ALDH1 (**c**) and CD44 (**d**) expression (10× and 40× magnification) in HNSCC. White bar in 10× magnification equals 100 µm, black bar in 40× magnification equals 20 µm. Samples from the cohort with high IHC scores regardless of HPV status were chosen. ALDH1, BCL11B, BMI-1, and CD44 staining is red-brown, nuclei and cytoplasm are counter-stained with hemalaun (blue).
